# Generative artificial intelligence reshapes university students' sustainable use of academic information resources: mixed evidence from behavioral logs and perceptual survey data

**DOI:** 10.3389/fpsyg.2026.2002741

**Published:** 2026-10-26

**Authors:** Zhenzhen Huang, Xiao Song, Jiayu Zhuang, Shuo Xiao

**Affiliations:** 1 Library, China University of Mining and Technology, Xuzhou, Jiangsu, China; 2 School of Computer Science and Technology/School of Artificial Intelligence, China University of Mining and Technology, Xuzhou, Jiangsu, China; 3 Agricultural Information Institute, Chinese Academy of Agricultural Sciences, Beijing, China

**Keywords:** academic information resources, academic libraries, auxiliary GenAI use, generative artificial intelligence, interrupted time-series, quantitative triangulation, substitutive GenAI use

## Abstract

**Introduction:**

Generative artificial intelligence (GenAI) is reshaping how students retrieve and consume academic information, yet whether it substitutes for or complements library resources remains contested, and prior surveys rarely separate auxiliary from substitutive use.

**Methods:**

This study uses a convergent triangulation design that combines multiple quantitative methods. It addresses the same set of research propositions by collecting perceptual-level questionnaire data and behavioral-level logs and platform usage statistics, analyzing them independently, and comparing the findings at the interpretation stage. Using 368 student questionnaires, we test an auxiliary-substitutive dual-path structural equation model and compare the results with an interrupted time-series analysis of 3.2 million print-loan logs (2017-2025) and digital-platform statistics from 2021 to the first half of 2026.

**Results:**

Print-resource continuance intention is positively associated with deep-reading demand, but not significantly associated with auxiliary GenAI use. Survey results showed auxiliary GenAI use positively associated with digital-resource continuance intention, yet insufficiently distinct from perceived digital-resource convenience; platform data showed nascent use of embedded AI features. Substitutive GenAI use shows only a weak negative association with print-resource continuance intention, while the nine-year loan record reveals no significant level or trend change at the January 2023 breakpoint; task-channel fit shows no significant moderating effect.

**Discussion:**

In this institution, findings align more with dual-channel coexistence than with the “AI-replaces-libraries” narrative; the survey-level substitution association has not yet appeared in aggregate borrowing behavior, informing collection development, service redesign, and AI-literacy education.

## 1 Introduction

Generative artificial intelligence (GenAI) is reshaping the production, retrieval, and consumption of academic information (Kasneci et al., 2023; Michel-Villarreal et al., 2023). Since the release of ChatGPT in 2022, AI tools have entered university students' learning processes both as standalone applications and as platform-embedded AI features—such as AI search, AI question answering, AI-assisted reading, and AI-assisted creation—integrated into academic database platforms such as China National Knowledge Infrastructure (CNKI) (Cox et al., 2019; Mannuru et al., 2024). For academic libraries, whose core mission is to ensure access to academic information resources, this technological transformation also bears directly on the provision of such resources. If students bypass library resources and turn to GenAI for information, the print collections and digital-resource systems developed by libraries over decades may be challenged. Conversely, if GenAI can improve resource utilization, libraries will need to redesign their service models to accommodate an ecosystem of print resources, digital resources, and AI. In either case, whether GenAI complements or substitutes for the use of academic information resources must be tested using empirical data. Beyond academic information seeking, recent research has also examined AI-guided applications in other student-facing domains, including AI-supported mental-health interventions (Wang et al., 2026). More generally, recent research on digitally mediated learning suggests that students' online learning experiences are shaped not only by the availability of digital technologies but also by social and self-regulatory factors, indicating that students' responses to technology-mediated learning environments may vary according to their learning-related characteristics and contextual conditions (Huang et al., 2025).

Existing research offers two opposing perspectives. The substitution perspective holds that GenAI can provide answers in a one-stop manner and may weaken students' reliance on library resources; surveys report a continued increase in the proportion of students who use AI as their primary information source (Kolawole et al., 2025; Abbas et al., 2024). The complementarity perspective, by contrast, argues that AI functions for search guidance, literature screening, and content comprehension can lower barriers to using academic resources and encourage further use of library resources (Jha, 2023; Huang, 2024). Although both perspectives are supported by survey evidence, they share several limitations. First, the evidence comes almost exclusively from self-administered questionnaires and lacks corroboration from behavioral data; research on information behavior shows that self-reported use and actual use may differ systematically. Second, most studies treat GenAI use as a single construct and do not distinguish auxiliary GenAI use, which involves using GenAI to locate and understand literature, from substitutive GenAI use, which involves directly using GenAI-generated content in place of literature reading. The two forms of use have opposite theoretical implications. Third, few studies have simultaneously examined academic information use in contexts where the three information channels of print resources, digital resources, and GenAI coexist (Trevisan et al., 2024; Abad-Segura et al., 2020; Shenkoya and Kim, 2023; Mohamed Hashim et al., 2022). The question, therefore, is not simply whether AI will replace libraries, but which resource-use structure can persist over time.

From an educational-psychology perspective, these questions concern how students regulate their learning in technology-rich environments. Self-regulated learning theory holds that learners actively select, monitor, and adjust the strategies and resources they use to achieve academic goals (Zimmerman, 2002). Accordingly, students' continuance intention to use print resources, continuance intention toward digital resources, and auxiliary and substitutive GenAI use can be understood through the lens of learning-related resource selection. Cognitive offloading offers a common point of departure for understanding the two forms of GenAI use: students draw on external tools to share information-acquisition and processing tasks (Risko and Gilbert, 2016), but the stages delegated to AI differ. Auxiliary use helps students retrieve, screen, and understand academic materials while still relying on their own reading and synthesis of the literature; substitutive use directly generates complete passages for papers, reports, or assignments, extending AI's role to the production of task-related text. Print-resource continuance intention can also be understood through deep-reading demand, which reflects students' need for sustained reading and systematic study. Accordingly, this study uses cognitive offloading and deep-reading demand as two perspectives for interpreting associations among the survey variables; print-loan records and digital-platform usage statistics describe institutional-level changes in resource use and provide context for interpreting the survey findings.

Using China University of Mining and Technology as a case, this study addresses these three limitations. It adopts a multimethod quantitative research design. Based on 368 valid questionnaire responses from university students, it develops an auxiliary-substitutive dual-path structural equation model (SEM) to examine the distinct associations of auxiliary and substitutive GenAI use with print-resource continuance intention and continuance intention toward digital resources. It also uses 3.2 million library circulation logs from 2017 to 2025 and platform usage statistics for the digital resource platform from 2021 to the first half of 2026 to examine, through interrupted time-series and trend analyses, points of convergence and divergence between survey-level associations and aggregate institutional resource-use trends with respect to the study's research propositions. The study addresses three research questions:

**RQ1**: How are auxiliary GenAI use and substitutive GenAI use each associated with university students' print-resource continuance intention and continuance intention toward digital resources?
**RQ2**: Does task–channel fit moderate the relationship between GenAI use and university students' continuance intention toward library resources?
**RQ3**: What patterns of convergence or divergence emerge when survey-level substitution and complementarity associations are compared with aggregate trends in print borrowing and digital-platform use? With January 2023 specified as the breakpoint, did print borrowing show an observable change in level or trend?

The study makes theoretical, methodological, and practical contributions. Theoretically, it proposes and tests an auxiliary-substitutive dual-path model that examines, within a single framework, the distinct associations of auxiliary and substitutive GenAI use with continuance intentions toward print and digital resources. Survey results showed a close association between perceived convenience of digital resources and auxiliary GenAI use, although the two constructs showed insufficient discriminant validity; platform statistics showed that AI features were embedded in digital-resource services, but their use remained at an early stage. Methodologically, the study triangulates millions of behavioral log records with questionnaire evidence and finds that the survey-level substitution association has not yet been reflected in aggregate borrowing behavior, thereby offering a point of reference for reducing the field's reliance on cross-sectional self-reported data. Practically, the study operationalizes the use of academic information resources in the GenAI era as testable empirical questions and provides evidence relevant to academic libraries' collection development, service redesign, and AI-literacy education across three channels: print resources, digital resources, and AI. This educational dimension is particularly important because research on digital behavior has linked Internet literacy with patterns of technology use among young people, highlighting the importance of developing informed and responsible digital-use capabilities (Jiang et al., 2023).

## 2 Theoretical foundations and research hypotheses

The four theoretical perspectives adopted in this study play distinct and complementary roles, and we make this division of labor explicit. Information ecology theory provides the overarching framework: it motivates treating print resources, digital resources, and GenAI as coexisting channels within a single information environment and underlies the path hypotheses that connect them. The expectation–confirmation model of information systems continuance (ECM-ISC) provides a perspective for understanding continuance intention toward print and digital resources, represented by CIP and CID, respectively. For the print channel, deep-reading demand provides the basis for H1. For the digital channel, H2 links perceived convenience of digital resources to auxiliary GenAI use; the convenience of digital platforms and the AI features embedded in them support this hypothesis and reflect the overlap between digital resources and AI functions within the information-ecology framework. Media substitution and complementarity theory generates the dual-path core of the model: it distinguishes auxiliary use, which improves the efficiency of using existing resources, from substitutive use, which replaces them, and thus yields the complementary paths H3 and H4 and the substitution paths H5 and H6. Finally, task–technology fit theory specifies the boundary condition: whether auxiliary use is converted into continued use depends on the fit between AI functions and task requirements (H7). Figure 1 presents the integrated conceptual model, in which each set of paths is annotated with the theoretical perspective from which it is derived.

**Figure 1:**
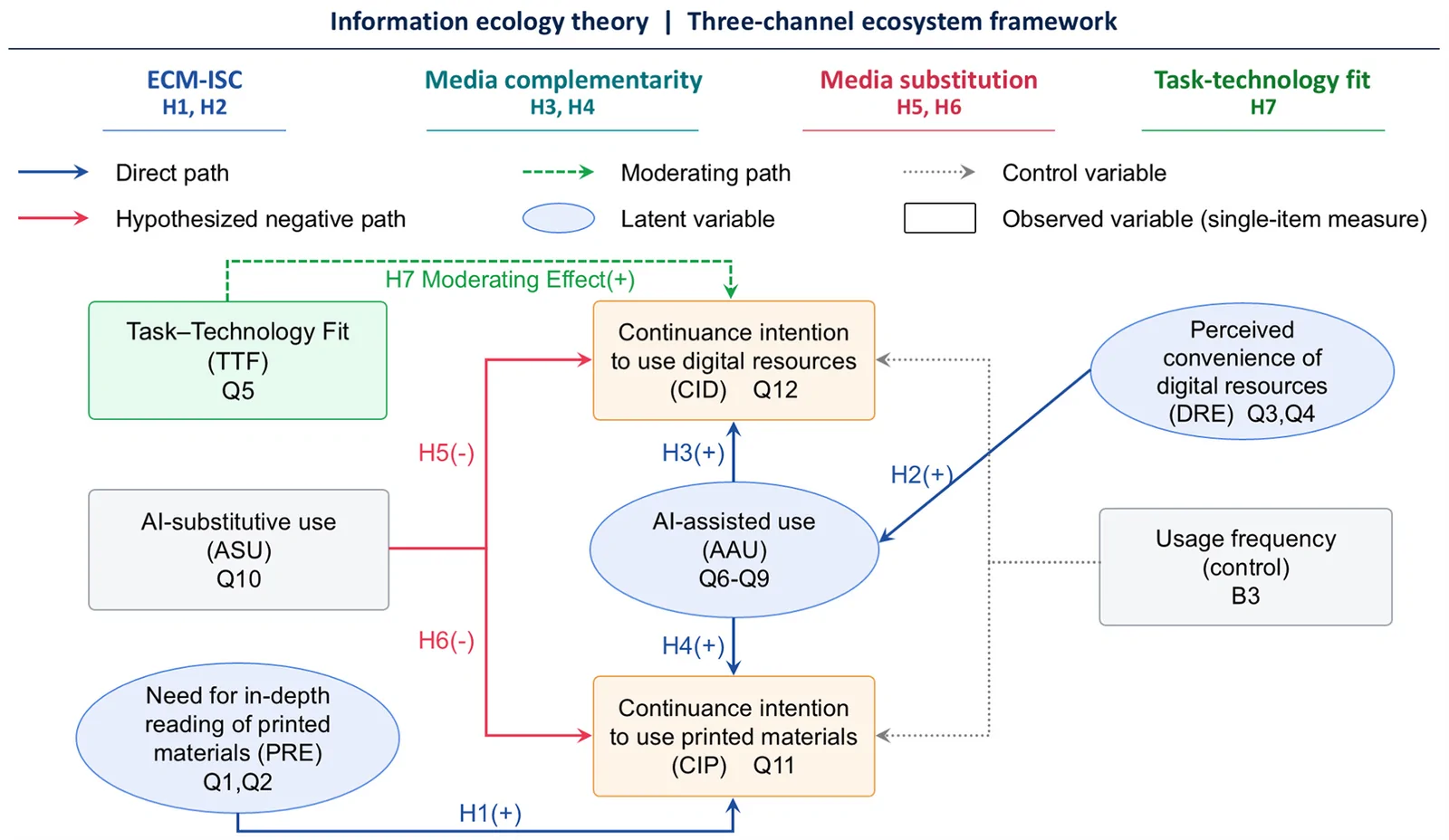
Conceptual model.

### 2.1 A three-channel information ecosystem

Information ecology theory views an information environment as a dynamic system jointly constituted by people, information, technology, and organizational practices (Nardi and O'Day, 1999). Different information channels do not engage in simple zero-sum competition; instead, they may compete, coexist, and evolve. University students currently obtain academic information through three main channels: a print channel represented by print books, a digital channel represented by academic databases, and an AI channel represented by GenAI tools. The three channels serve different scenarios and functions. The print channel suits sustained reading and systematic study; the digital channel facilitates rapid retrieval and bulk acquisition of literature; and the AI channel provides conversational, generative information processing. In using academic information resources, the three channels, print resources, digital resources, and GenAI, do not simply substitute for one another; rather, they compete with and complement one another, and each has its own basis for continued use. Drawing on information ecology theory, this study operationalizes the relationships between GenAI and different academic information resource channels as testable path hypotheses.

### 2.2 Continuance intention toward resources

The expectation-confirmation model of information systems continuance (ECM-ISC) (Bhattacherjee, 2001) posits that users' willingness to continue using an information resource or technology depends on their prior experience with it and the degree to which it fits their needs.

For the print channel, deep-reading demand is an important factor in understanding print-resource continuance intention. Previous research suggests that print media have certain advantages for reading comprehension and cognitive-load management (Mangen et al., 2013; Baron, 2017; Delgado et al., 2018; Clinton, 2019; Jian, 2022). These advantages may be especially relevant to tasks that require sustained attention and systematic learning. Therefore, stronger deep-reading demand may be associated with stronger continuance intention to use print resources. Accordingly, the following hypothesis is proposed:

**H1**: Deep-reading demand for print resources is positively associated with university students' intention to continue using print resources.

For the digital channel, this study focuses on the association between perceived convenience of digital resources and auxiliary GenAI use. Such convenience is mainly reflected in rapid retrieval, access across time and space, and bulk downloading. Students can access AI search, AI question answering, and AI-assisted reading through standalone AI tools and increasingly embedded features on digital-resource platforms. The more students recognize the convenience of digital platforms, the more likely they are to encounter, try, and gradually become accustomed to using the platform-embedded AI features. Perceived convenience of digital resources therefore provides a basis for auxiliary GenAI use. Accordingly, the following hypothesis is proposed:

**H2**: Perceived convenience of digital resources is positively associated with university students' auxiliary GenAI use.

### 2.3 AI assistance and substitution

Media substitution and complementarity theory provides a theoretical basis for distinguishing the two forms of AI use (Dutta-Bergman, 2004; Dimmick et al., 2004; Lee and Leung, 2008; Lin, 2004). The theory holds that the effect of a new medium on an existing medium depends on the extent to which their functions overlap. Substitution occurs when a new medium assumes the core functions of an existing medium, whereas complementarity occurs when it improves the efficiency of using an existing medium or expands its use scenarios. Accordingly, the effects of GenAI on academic information resources can be divided into an auxiliary path and a substitutive path.

#### 2.3.1 Auxiliary path (complementarity)

Auxiliary GenAI use refers to using AI tools or platform-embedded AI features for literature retrieval and screening, content summarization, concept explanation, and comparison of viewpoints. Auxiliary use supports the retrieval, screening, and comprehension of academic materials through standalone AI tools or platform-embedded functions, whereas substitutive use centers on directly generating text for academic tasks. Items Q6–Q9 capture distinct auxiliary functions, and Q10 captures the direct generation of complete passages for papers, reports, or assignments (Supplementary Table S1). We treat this direct generation of passages as a concrete form of substitutive use; the same student may use both forms for different tasks. Auxiliary use does not directly generate complete passages for academic tasks; rather, it lowers the cost of discovering academic resources and the threshold for understanding them. In this process, AI guides and supports the use of digital resources, making retrieval more precise, screening more efficient, and understanding deeper, thereby strengthening students' reliance on digital resource platforms and their continuance intention toward digital resources. Accordingly, the following hypothesis is proposed:

**H3**: Auxiliary GenAI use is positively associated with university students' continuance intention toward digital resources.

The effect of auxiliary GenAI use on the print channel is theoretically uncertain. On the one hand, auxiliary GenAI use may shift students' overall information activities to digital environments, thereby competing with print reading for time. On the other hand, AI-guided literature discovery may spill over into the print collection. This study follows the complementarity logic and proposes:

**H4**: Auxiliary GenAI use is positively associated with university students' print-resource continuance intention.

#### 2.3.2 Substitutive path

Substitutive GenAI use, in this context, refers to directly generating complete passages for papers, reports, or assignments. When students bypass the processes of searching, reading, and analysis to obtain finished content directly, the value of using library resources is partially displaced. Unlike the auxiliary path, substitutive GenAI use weakens reliance on resources themselves and is expected to negatively affect both print-resource continuance intention and continuance intention toward digital resources. Accordingly, the following hypotheses are proposed:

**H5**: Substitutive GenAI use is negatively associated with university students' continuance intention toward digital resources.
**H6**: Substitutive GenAI use is negatively associated with university students' print-resource continuance intention.

### 2.4 The moderating role of task–channel fit

Task–technology fit (TTF) theory (Goodhue and Thompson, 1995) holds that whether technology can be converted into actual performance and continued use depends on the fit between technological functions and task requirements. In this ecosystem, students' ability to select and switch appropriately among print resources, digital resources, and AI channels based on learning-task characteristics determines whether auxiliary GenAI use can effectively translate into continuance intention toward digital resources. Students with stronger task–channel fit are better able to embed AI features for retrieval guidance and content processing into appropriate task contexts, and the complementary effect should therefore be stronger. Conversely, students with weaker fit may generalize AI as an all-purpose tool, making it more difficult for its auxiliary value to be realized. Accordingly, the following hypothesis is proposed:

**H7**: Task–channel fit positively moderates the association between auxiliary GenAI use and continuance intention toward digital resources, such that the positive H3 association is stronger when task–channel fit is higher.

Figure 1 presents the conceptual model, in which each set of paths is annotated with the theoretical perspective from which it is derived. It treats deep-reading demand for print resources, perceived convenience of digital resources, auxiliary GenAI use, and substitutive GenAI use as inputs; print-resource continuance intention and continuance intention toward digital resources as outcomes; task–channel fit as the moderator; and use frequency as a control variable. In the testing strategy, questionnaire data assess students' use intentions at the perceptual level, whereas behavioral logs and platform usage statistics assess corresponding aggregate behavior. The print-loan records are used to examine changes in aggregate borrowing trends after 2023, while platform statistics describe trends in traditional digital-resource use and platform-embedded AI features. These aggregate trends can be compared with the survey findings but cannot validate individual-level paths. The triangulation of the two forms of evidence is central to the study's multimethod quantitative research design.

## 3 Research design and methods

### 3.1 Research design

This study uses a convergent triangulation design that combines multiple quantitative methods. It collects questionnaire data at the perceptual level and behavioral logs and platform usage statistics at the behavioral level for the same set of research propositions, analyzes them independently, and triangulates them during interpretation. The design is based on the following logic: questionnaire data can measure psychological constructs such as use intention, perception, and motivation, but are susceptible to social-context effects and self-report bias; large-scale behavioral data objectively record actual use trajectories, but cannot reveal the mechanisms underlying behavior. The complementary combination of the two forms of data enables the study to analyze survey-level associations in resource use (RQ1 and RQ2) and compare aggregate institutional resource-use trends with the corresponding research propositions to identify points of convergence and divergence (RQ3). The study proceeds through measurement-model assessment, structural-model assessment, aggregate contextual triangulation of the behavioral data, and cross-level interpretation. Because the survey respondents cannot be individually linked to the institutional logs under the institution's data-governance rules, the behavioral data provide aggregate contextual corroboration for the research propositions addressed by the survey, but cannot validate individual-level SEM paths; convergence between the two sources is therefore assessed at the level of research propositions and is not extended to individual behavior. Recent computational research has also demonstrated the increasing use of large-scale digital usage data and language-model-based approaches for representing application-use behavior, further illustrating the growing methodological importance of behavioral digital traces in technology research (Huang et al., 2026).

### 3.2 Survey data sources and variable measurement

The data were collected via a questionnaire survey of enrolled students at China University of Mining and Technology in 2026. The questionnaire is provided in Supplementary Table S1 and was distributed online, yielding 368 valid questionnaire responses. Table 1 reports the definitions and descriptive statistics of the main variables. This study designed questionnaire items around university students' use of and continuance intentions toward print resources, digital resources, and GenAI tools. Item development drew on conceptual definitions from research on continuance intention, media complementarity, and task–technology fit (Bhattacherjee, 2001; Dutta-Bergman, 2004; Goodhue and Thompson, 1995), with wording tailored to university learning and academic-information use. The complete items and response coding are reported in Supplementary Table S1. Deep-reading demand, perceived convenience of digital resources, and auxiliary GenAI use were measured with multiple items (Cronbach's α = 0.826–0.908). Substitutive GenAI use (ASU), task–channel fit (TTF), print-resource continuance intention (CIP), and digital-resource continuance intention (CID) were each measured with a single item. Specifically, Q10 asks whether respondents directly use AI tools to generate complete passages for papers, reports, or assignments; Q5 asks whether they choose among print resources, digital resources, and AI tools according to task needs; and Q11 and Q12, respectively, ask whether they intend to continue using print and digital resources even when AI tools are available (Supplementary Table S1). These items focus on the specific behaviors and intentions examined in this study. The single-item measurement literature offers a conditional rationale for this choice (Bergkvist and Rossiter, 2007), but it does not establish that all four constructs have been adequately measured. Accordingly, we interpret the corresponding paths as associations between the specific behaviors and intentions represented by these items.

**Table 1:** Definitions and descriptive statistics of the main variables.

Variable	Definition	Mean	Standard deviation	Minimum	Maximum
Dependent variables					
Print-resource continuance intention (CIP)	Respondents' strength of intention to continue using library print books in the future; measured by single item Q11; 1 = strongly disagree, 5 = strongly agree	3.772	1.200	1.000	5.000
Digital-resource continuance intention (CID)	Respondents' strength of intention to continue using the library's digital resource platform in the future; measured by single item Q12; 1 = strongly disagree, 5 = strongly agree	3.815	1.167	1.000	5.000
Independent variables					
Deep-reading demand for print resources (PRE)	Respondents' demand for print books in intensive or systematic reading scenarios; mean of Q1 and Q2; 1 = strongly disagree, 5 = strongly agree	3.697	1.187	1.000	5.000
Perceived convenience of digital resources (DRE)	Respondents' evaluation of the convenience of the digital resource platform for rapid retrieval and access across time and space; mean of Q3 and Q4; 1 = strongly disagree, 5 = strongly agree	3.950	1.126	1.000	5.000
Auxiliary GenAI use (AAU)	Extent to which respondents use AI tools or platform-embedded AI features to support search and screening, content summarization, lead identification, and comparison of viewpoints; mean of Q6–Q9; 1 = strongly disagree, 5 = strongly agree	3.901	1.040	1.000	5.000
Substitutive GenAI use (ASU)	Extent to which respondents directly use AI tools to generate complete paragraphs for papers, reports, or assignments; measured by single item Q10; 1 = strongly disagree, 5 = strongly agree	3.486	1.291	1.000	5.000
Moderator					
Task–channel fit (TTF)	Extent to which respondents select among print books, digital resources, and AI tools according to their needs in different learning tasks; measured by single item Q5; 1 = strongly disagree, 5 = strongly agree	3.875	1.168	1.000	5.000
Control variable					
Use frequency (B3)	Frequency of using library print or digital resources in the previous semester; 1 = almost every day, 2 = 3–5 times per week, 3 = 1–2 times per week, 4 = 2–3 times per month, 5 = once per month or less	3.027	1.293	1	5
Demographic background variables					
Student status (B1)	Respondents' student status; 1 = undergraduate student, 2 = master's student, 3 = doctoral student, 4 = other	1.307	0.609	1	4
Discipline category (B2)	Respondents' disciplinary category; 1 = humanities and social sciences, 2 = science and engineering, 3 = agriculture and medicine, 4 = economics, management, and law, 5 = arts and sports, 6 = other	2.337	1.179	1	6

### 3.3 Behavioral logs and platform usage statistics

#### 3.3.1 Library circulation logs

The data were exported from the integrated management system of the China University of Mining and Technology Library and comprise borrowing, return, and renewal transaction logs from 2017 to 2025, totaling approximately 3.2 million records. The observation period captures a long borrowing trend before the emergence of GenAI and includes three complete years from 2023 to 2025, allowing comparison of the existing trend with changes after the prespecified breakpoint. Each record includes fields such as transaction time, transaction type, call number, document type, reader type, department, library-card number, and borrowing, due, and return dates. Before analysis, library-card numbers were hashed, and no personally identifiable information was retained in the dataset; only aggregate-level indicators were used in the analysis. Core indicators included monthly and annual loan volume, return volume, renewal volume, the annual number of unique borrowers, annual loans per borrower, and loan composition by reader type. The results are reported in Table 2.

**Table 2:** Annual indicators of print-book circulation.

Year	Loan volume	Return volume	Renewal volume	Unique borrowers	Annual loans per borrower	Undergraduate loans	Master's loans	Doctoral loans	Other loans
2017	406,120	412,119	0	30,033	13.522	292,600	71,977	10,081	31,462
2018	348,830	357,574	0	27,753	12.569	240,092	66,937	10,104	31,697
2019	266,163	268,095	1,046	25,037	10.631	165,874	56,890	9,339	34,060
2020	95,212	106,397	7,999	14,488	6.572	54,075	25,958	3,970	11,209
2021	134,739	137,285	13,010	15,700	8.582	63,477	31,897	5,474	33,891
2022	81,213	88,126	13,670	11,043	7.354	39,769	21,571	3,292	16,581
2023	93,054	92,849	16,117	11,877	7.835	47,461	23,145	4,925	17,523
2024	80,027	82,979	15,260	9,900	8.084	32,581	17,323	4,450	25,673
2025	46,339	48,863	14,205	7,278	6.367	21,852	9,686	3,240	11,561

#### 3.3.2 Digital resource platform usage statistics

The data were drawn from usage-statistics reports for the library's digital resource management platform from 2021 through the first half of 2026 and cover product, subject, and account dimensions. This study uses user operations at the product dimension as indicators, including traditional operations, AI-feature operations, and the proportion of AI operations. The results are reported in Table 3.

**Table 3:** Annual trends in traditional operations and AI-feature operations on the digital resource platform.

Year	Traditional operations (search + browse + download)	AI-feature operations	Total operations	Share of AI operations (%)
2021	279,834,736	0	279,834,736	0
2022	272,526,830	0	272,526,830	0
2023	234,404,671	0	234,404,671	0
2024	282,131,877	0	282,131,877	0
2025	317,332,412	326,449	317,658,861	0.1
First half of 2026	144,063,539	766,246	144,829,785	0.53

AI-feature operations = AI search + AI question answering + AI-assisted reading + AI-assisted creation + AI download/numerical-value download + AI achievements.

#### 3.3.3 Interruption-point specification

The interrupted time-series model includes two prespecified breakpoints: February 2020 and January 2023. February 2020 marks the COVID-19 outbreak period, when campus closure caused on-site borrowing to halt abruptly. Following ChatGPT's release in late November 2022, we prespecified January 2023 as a reference breakpoint to examine the early diffusion of GenAI; this analytical specification is not intended to establish the date of GenAI adoption among students at this university. Post-interruption trend terms begin in the month after each interruption point. Local platform statistics first recorded operations involving platform-embedded AI features in 2025 (Table 3). We acknowledge that the end of COVID-19-related campus restrictions around the same period is a competing event; the model includes an explicit COVID-19 interruption, but residual confounding from concurrent institutional or policy changes cannot be fully excluded. To examine whether the null post-2023 findings depend on the chosen breakpoint, we re-estimated the model with three alternative interruptions—March 2023, September 2023, and January 2025 (the start of the first year in which local platform statistics recorded AI-feature operations). Under all alternative specifications, the post-interruption level and slope changes remained statistically non-significant (Supplementary Table S2). The decline in print borrowing did not significantly accelerate after the diffusion of GenAI, and this finding was consistent across alternative breakpoint specifications.

### 3.4 Analytical strategy

#### 3.4.1 Measurement-model assessment

For multi-item constructs, reliability is assessed using Cronbach's α and composite reliability (CR), with values above 0.70 as the criterion. Convergent validity is assessed using standardized factor loadings and average variance extracted (AVE). Standardized factor loadings should exceed 0.70, and AVE should exceed 0.50. Discriminant validity is assessed using the Fornell–Larcker criterion (Fornell and Larcker, 1981) and the heterotrait–monotrait ratio (HTMT) (Henseler et al., 2015), with 0.85 used as a strict HTMT benchmark. For the eight items measuring PRE, DRE, and AAU, the three-factor model is compared with a two-factor model that combines DRE and AAU while retaining PRE as a separate factor, as well as a one-factor model. Model fit is compared using χ^2^, degrees of freedom, CFI, TLI, and RMSEA. A 95% percentile confidence interval for the DRE–AAU HTMT is estimated from 2,000 bootstrap resamples. Single-item variables are not included in these reliability and validity assessments. Common method bias (CMB) is preliminarily diagnosed using Harman's single-factor test (Podsakoff et al., 2003). Full collinearity variance inflation factor (full collinearity VIF) is used to assess the risk of collinearity in the model, with VIF values expected to be below 3.3 (Kock, 2015). Because Harman's single-factor test provides only a preliminary assessment and VIF is primarily a collinearity diagnostic, the associations among questionnaire variables are interpreted with potential common method variance in mind.

#### 3.4.2 Structural-model assessment

Covariance-based structural equation modeling (CB-SEM) is used to estimate the hypothesized paths. Following the theoretically specified DRE → AAU → CID path, the model estimates the indirect association between DRE and CID via AAU. Standardized path coefficients and the 95% confidence interval (95% CI) for the indirect effect are computed using 500 bootstrap resamples. The analysis reports *f*^2^ effect sizes and model fit indices. Values of 0.02, 0.15, and 0.35 for *f*^2^ effect sizes indicate small, medium, and large effects, respectively. Model fit indices include χ^2^/*df*, comparative fit index (CFI), Tucker–Lewis index (TLI), and root mean square error of approximation (RMSEA). To test the moderating effect, AAU construct scores and TTF scores are mean-centered before constructing the interaction term (AAU × TTF). The corresponding equations include the main effect of TTF, the interaction term, and use frequency (B3).

### 3.5 Model equations

#### 3.5.1 Structural equation model (SEM)

Based on the preceding theoretical reasoning and research hypotheses, the structural model is specified as follows:

$$\begin{array}{l} AAU_{i}=\alpha_{0}+\alpha_{1}DRE_{i}+\varepsilon_{1i} \end{array}$$ (1)

$$\begin{array}{l} CIP_{i}=\beta_{0}+\beta_{1}PRE_{i}+\beta_{2}AAU_{i}+\beta_{3}ASU_{i}+\beta_{4}B3_{i}+\varepsilon_{2i} \end{array}$$ (2)

$$\begin{array}{l} CID_{i}=\gamma_{0}+\gamma_{1}AAU_{i}^{c}+\gamma_{2}ASU_{i}+\gamma_{3}TTF_{i}^{c} \end{array}$$

$$\begin{array}{l} +\gamma_{4}\left(AAU_{i}^{c}\times TTF_{i}^{c}\right)+\gamma_{5}B3_{i}+\varepsilon_{3i} \end{array}$$ (3)

In these equations, subscript *i* denotes respondent *i*, where *i* = 1, …, 368. DRE denotes perceived convenience of digital resources; AAU denotes auxiliary GenAI use; ASU denotes substitutive GenAI use; PRE denotes deep-reading demand for print resources; TTF denotes task–channel fit; CIP and CID denote print-resource continuance intention and digital-resource continuance intention, respectively; and B3 denotes the use-frequency control variable. To test the moderating role of task–channel fit, AAU and TTF are first mean-centered, defined as $AAU_{i}^{c}=AAU_{i}$-$\bar{AAU}$ and $TTF_{i}^{c}=TTF_{i}$-$\bar{TTF}$, and the interaction term $AAU_{i}^{c}\times TTF_{i}^{c}$ is then constructed, where superscript *c* denotes a mean-centered variable. Parameters α_0_, β_0_, and γ_0_ are intercepts; α_1_, β_1_ to β_4_, and γ_1_ to γ_5_ are coefficients to be estimated; and ε_1*i*_, ε_2*i*_, and ε_3*i*_ are stochastic disturbance terms. Equation 1 estimates the path association between DRE and AAU and corresponds to H2. Equation 2 uses CIP as the dependent variable and estimates its path associations with PRE, AAU, and ASU, corresponding to H1, H4, and H6. Equation 3 uses CID as the dependent variable and estimates its path associations with AAU, ASU, TTF, and the interaction between *AAU*^*c*^ and *TTF*^*c*^. The paths of AAU and ASU correspond to H3 and H5, respectively; the interaction term corresponds to H7; and the main effect of TTF is included as a lower-order term in the moderation model. B3 is included as a control variable in Equations 2, 3.

When task–channel fit is at the sample mean, $TTF_{i}^{c}=0$, the conditional indirect effect of DRE on CID is calculated using the product-of-coefficients method (Equation 4):

$$\begin{array}{l} Indirect\left(\text{DRE}\to \text{CID}|TTF_{i}^{c}=0\right)=\alpha_{1}\times \gamma_{1} \end{array}$$ (4)

Here, α_1_ and γ_1_ are the estimated coefficients for the DRE–AAU and AAU–CID paths in Equations 1, 3, respectively. When task–channel fit is at its sample mean, $TTF_{i}^{c}=0$ and the interaction between AAU and TTF is also zero. Under this condition, γ_1_ represents the path association between AAU and CID, whereas α_1_×γ_1_ is the model-specified estimate of the indirect association between DRE and CID via AAU. This study uses 500 bootstrap resamples to calculate the 95% confidence interval for this indirect effect. The conditional indirect effect is considered significant when the 95% confidence interval excludes zero.

#### 3.5.2 Interrupted time-series model (ITS)

An interrupted time-series model is constructed with interruption points in February 2020 and January 2023 (Bernal et al., 2017) (Equation 5):

$$\begin{array}{l} Y_{t}=\delta_{0}+\delta_{1}T_{t}+\delta_{2}D_{t}^{2020}+\delta_{3}T_{t}^{2020}+\delta_{4}D_{t}^{2023}+\delta_{5}T_{t}^{2023} \end{array}$$

$$\begin{array}{l} +\overset{12}{\underset{m=2}{\sum }}\theta_{m}M_{mt}+\varepsilon_{t} \end{array}$$ (5)

Here, subscript *t* denotes the month included in the ITS regression, ranging from 1 to 107. The analytic period covers January 2017 to December 2025. No borrowing record was available for February 2020, so that month was excluded from the regression estimation. *Y*_*t*_ denotes monthly print-book loan volume in month *t*, and *T*_*t*_ is the time-trend term. $D_{t}^{2020}$ and $D_{t}^{2023}$ are interruption indicators that take the value 1 from the COVID-19 interruption point in February 2020 and the GenAI interruption point in January 2023, respectively, and 0 before the corresponding interruption point. Their coefficients, δ_2_ and δ_4_, capture level changes at the interruption points. $T_{t}^{2020}$ and $T_{t}^{2023}$ are post-interruption trend terms. They take the value of 0 in the interruption month, increase to 1 in the following month, and are 0 before the interruption point. Their coefficients, δ_3_ and δ_5_, capture post-interruption slope changes. *M*_*mt*_ denotes month dummy variables, with January as the reference group, to control for semester seasonality. δ_0_ is the intercept and represents the baseline level; δ_1_ is the baseline trend; and ε_*t*_ is the disturbance term. Newey–West heteroskedasticity- and autocorrelation-consistent (HAC) standard errors are used, with a lag length of 12.

## 4 Results

### 4.1 Measurement model: reliability and validity

The original scale was coded from 1 = strongly agree to 5 = strongly disagree. To ensure that higher scores indicated greater agreement, all items were reverse-scored, so that 1 = strongly disagree and 5 = strongly agree. Reliability testing showed that Cronbach's α values for deep-reading demand for print resources (PRE; Q1–Q2), perceived convenience of digital resources (DRE; Q3–Q4), and auxiliary GenAI use (AAU; Q6–Q9) were 0.826, 0.836, and 0.908, respectively. All exceeded the conventional threshold of 0.70, as shown in Table 4.

**Table 4:** Summary of reliability and validity.

Construct	Items	No. of items	Mean	SD	Cronbach's α	CR	AVE
Deep-reading demand for print resources (independent variable)	Q1, Q2	2	3.697	1.187	0.826	0.828	0.707
Perceived convenience of digital resources (independent variable)	Q3, Q4	2	3.950	1.126	0.836	0.837	0.720
Auxiliary GenAI use (independent variable)	Q6, Q7, Q8, Q9	4	3.901	1.040	0.908	0.909	0.713
Substitutive GenAI use (independent variable)	Q10	1	3.486	1.291	–	–	–
Task–channel fit (moderator)	Q5	1	3.875	1.168	–	–	–
Print-resource continuance intention (dependent variable)	Q11	1	3.772	1.200	–	–	–
Digital-resource continuance intention (dependent variable)	Q12	1	3.815	1.167	–	–	–

*N* = 368; SD, standard deviation; Cronbach's α, CR, and AVE are not calculated for single-item variables.

A confirmatory factor analysis (CFA) was conducted for the eight items included in PRE, DRE, and AAU. The three-factor measurement model showed good fit: χ^2^(17) = 28.078, χ^2^/*df* = 1.65, CFI = 0.995, TLI = 0.992, and RMSEA = 0.042. The standardized factor loadings were 0.851 (Q1) and 0.831 (Q2) for PRE; 0.858 (Q3) and 0.839 (Q4) for DRE; and 0.814 (Q6), 0.871 (Q7), 0.835 (Q8), and 0.856 (Q9) for AAU. All eight loadings exceeded 0.80. Composite reliability for the three dimensions ranged from 0.828 to 0.909, and average variance extracted (AVE) ranged from 0.707 to 0.720. All values met the recommended criteria, indicating satisfactory convergent validity.

Discriminant-validity results are shown in Tables 5, 6. The Fornell–Larcker criterion indicated that the latent-variable correlation between PRE and DRE was 0.783 and that between PRE and AAU was 0.766; both values were lower than the $\sqrt{\text{AVE}}$ for the relevant constructs, satisfying the criterion. The latent-variable correlation between DRE and AAU was 0.967, exceeding the $\sqrt{\text{AVE}}$ for DRE and AAU (0.849 and 0.844, respectively); therefore, the Fornell–Larcker criterion was not met for these two constructs. The three-factor model fit better than the one-factor model. The one-factor model had χ^2^(20) = 127.761, CFI = 0.951, TLI = 0.931, and RMSEA = 0.121; the difference from the three-factor model was statistically significant, with Δχ^2^ = 99.682, Δ*df* = 3, and *p* < 0.001. The two-factor model combining DRE and AAU while retaining PRE as a separate factor also fit well, with χ^2^(19) = 34.605, CFI = 0.993, TLI = 0.989, and RMSEA = 0.047. Although the three-factor model fit slightly better, this result alone does not establish adequate separation between DRE and AAU in this sample. The HTMT ratios were 0.786 for PRE and DRE and 0.765 for PRE and AAU, both below 0.85. For DRE and AAU, HTMT was 0.968, with a 95% bootstrap confidence interval of [0.912, 1.015] based on 2,000 resamples; even its lower bound exceeded 0.85. Thus, DRE and AAU were highly correlated in this sample and showed insufficient discriminant validity. For common method bias (CMB), the first factor in Harman's single-factor test explained 64.4% of the variance, exceeding the 40% threshold and indicating that the risk of common method bias cannot be ruled out. Across the structural equations, the maximum VIF of the predictors was 2.98, below the criterion of 3.3 (Kock, 2015), indicating no serious multicollinearity in the model. Two measurement issues deserve explicit comment. First, the very high DRE–AAU correlation (0.967) indicates that this sample did not empirically distinguish the two constructs. Accordingly, the DRE → AAU path is interpreted as a statistical association between two highly correlated constructs. The magnitude of its coefficient does not directly indicate the strength of a theoretical mechanism, and interpretation of the corresponding indirect effect is likewise constrained by construct overlap. The two-factor alternative also showed good fit, and the DRE–AAU HTMT interval remained above 0.85; thus, the three-factor model's better fit did not resolve the substantial overlap between these constructs. Second, the first factor in Harman's test explained 64.4% of the variance, so common method bias cannot be ruled out. The questionnaire did not request direct personal identifiers, and predictor and outcome items were presented separately; nevertheless, these arrangements do not exclude common method bias, and the DRE–AAU association may partly reflect method variance.

**Table 5:** HTMT matrix for discriminant validity.

Construct	PRE	DRE	AAU
PRE	1	0.786	0.765
DRE	0.786	1	0.968
AAU	0.765	0.968	1

**Table 6:** Fornell–Larcker discriminant validity assessment.

Construct	PRE	DRE	AAU
PRE	0.841	0.783	0.766
DRE	0.783	0.849	0.967
AAU	0.766	0.967	0.844

### 4.2 Structural model and hypothesis testing

DRE and AAU were highly correlated in this sample and did not meet the discriminant-validity criteria. The structural model retains the theoretically proposed DRE → AAU → CID path to examine associations among these variables under this model specification; this specification does not, by itself, eliminate the measurement overlap between DRE and AAU. Table 7 shows that the structural model, which included use frequency (B3) in the CIP and CID equations, demonstrated good fit: χ^2^ = 119.581, *df* = 54, χ^2^/*df* = 2.214, CFI = 0.981, TLI = 0.972, and RMSEA = 0.058. Student status (B1) and discipline (B2) were reported descriptively in Table 1 but were not included as covariates in this model. The explained variances (*R*^2^) for auxiliary GenAI use, print-resource continuance intention, and continuance intention toward digital resources were 0.986, 0.777, and 0.610, respectively.

**Table 7:** Model fit and diagnostic indices.

Indicator	Value	Criterion
χ^2^(*df*)	119.581 (54)	–
χ^2^/*df*	2.214	< 3
CFI	0.981	>0.90
TLI	0.972	>0.90
RMSEA	0.058	< 0.08
*R*^2^ (auxiliary GenAI use, AAU)	0.986	–
*R*^2^ (print-resource continuance intention, CIP)	0.777	–
*R*^2^ (digital-resource continuance intention, CID)	0.610	–
Full collinearity VIF (maximum in CIP equation)	2.070	< 3.3
Full collinearity VIF (maximum in CID equation)	2.983	< 3.3
Bootstrap resampling	500 successful resamples	–

The hypothesis-testing results are reported in Table 8. Deep-reading demand was positively associated with print-resource continuance intention. Deep-reading demand for print resources had a standardized path coefficient of 0.908 for print-resource continuance intention, *p* < 0.001, a 95% CI of [0.756, 1.136], and an *f*^2^ effect size of 0.949. H1 was supported, and the *f*^2^ value met the prespecified criterion for a large effect. This finding indicates a statistically significant association between deep-reading demand and print-resource continuance intention in this sample, but the magnitude of the path coefficient alone does not quantify the strength of an underlying psychological mechanism.

**Table 8:** Structural model hypothesis-testing results.

Hypothesis	Path	Expected direction	Standardized β	S.E.	*z*	*p*	Bootstrap 95% CI	*f* ^2^	Conclusion
H1	Deep-reading demand for print resources → print-resource continuance intention	+	0.908	0.100	9.062	< 0.001	[0.756, 1.136]	0.949	Supported
H2	Perceived convenience of digital resources → auxiliary GenAI use	+	0.993	0.052	18.976	< 0.001	[0.968, 1.000]	70.535	Supported
H3	Auxiliary GenAI use → digital-resource continuance intention	+	0.844	0.083	10.208	< 0.001	[0.652, 1.026]	0.490	Supported
H4	Auxiliary GenAI use → print-resource continuance intention	+	0.038	0.079	0.481	0.631	[–0.188, 0.190]	0.036	Not supported
H5	Substitutive GenAI use → digital-resource continuance intention	−	–0.030	0.041	–0.723	0.470	[–0.113, 0.057]	0.005	Not supported
H6	Substitutive GenAI use → print-resource continuance intention	−	–0.100	0.044	–2.250	0.024	[–0.187, –0.020]	0.104	Supported
H7	Auxiliary GenAI use × task–channel fit → digital-resource continuance intention	+	0.053	0.074	0.717	0.473	[–0.158, 0.270]	0.036	Not supported
H2·H3	Perceived convenience of digital resources → auxiliary GenAI use → digital-resource continuance intention (model-specified indirect association)	+	0.838	–	–	–	[0.641, 1.025]	–	Exploratory

H2 is supported only as a significant positive association under the specified three-factor model. Because DRE and AAU did not meet the discriminant-validity criterion, the indirect association should not be taken as evidence that a mediating mechanism has been established.

The digital-channel estimates showed close associations among the variables. Perceived convenience of digital resources was positively associated with auxiliary GenAI use, with a path coefficient of 0.993, *p* < 0.001, and a 95% CI of [0.968, 1.000], consistent with the expected direction of H2. The path coefficient from auxiliary GenAI use to continuance intention toward digital resources was 0.844, *p* < 0.001, with a 95% CI of [0.652, 1.026] and an *f*^2^ effect size of 0.490, supporting H3. In the three-factor model, the estimated indirect association between DRE and CID via AAU was 0.838, with a 95% CI of [0.641, 1.025], consistent with the indirect path specified in the model. The two-factor model combining DRE and AAU also fit well, with an HTMT of 0.968 between the constructs. Therefore, values such as β = 0.993 and *R*^2^ = 0.986 should be interpreted in light of their measurement overlap rather than taken, on their own, as indicators of the strength of a theoretical mechanism. The survey results indicate that digital resources and auxiliary GenAI use are closely associated in students' self-reports; platform statistics show that AI features have been embedded in digital resource platforms and that their use has increased. These findings are consistent with an interpretation of early-stage digital–AI channel integration, but the platform data cannot independently validate individual-level path relationships.

In this model, substitutive GenAI use showed a statistically significant but weak negative association only with print-resource continuance intention. Substitutive GenAI use was measured by Q10, which captures the direct use of GenAI to generate complete passages for papers, reports, or assignments. It showed a statistically significant negative association with print-resource continuance intention, β = −0.100, *p* = 0.024, 95% CI = [–0.187, –0.020], and an *f*^2^ effect size of 0.104. H6 was supported, but the effect size was small. Its association with digital-resource continuance intention was not statistically significant, β = −0.030 and *p* = 0.470, so H5 was not supported. Auxiliary GenAI use showed no statistically significant association with print-resource continuance intention, β = 0.038 and *p* = 0.631; H4 was not supported.

Task–channel fit was measured by Q5. The interaction between auxiliary GenAI use and task–channel fit did not significantly moderate digital-resource continuance intention, β = 0.053 and *p* = 0.473; H7 was not supported. This indicates that no statistically significant moderation by task–channel fit of the association between auxiliary GenAI use and digital-resource continuance intention was detected in this sample. The division of use across the three channels may reflect relatively stable patterns of use rather than differences in task–channel fit. Because substitutive GenAI use (ASU), task–channel fit (TTF), print-resource continuance intention (CIP), and digital-resource continuance intention (CID) were each measured with a single item, the H4–H7 findings should be interpreted in relation to the specific behaviors and intentions represented by those items, rather than as comprehensive tests of the corresponding constructs.

### 4.3 Behavioral logs and resource-use trends

#### 4.3.1 Trends in print borrowing and interruption-point tests

From 2017 to 2025, annual print-book loan volume at the university decreased from 406,120 to 46,339 loans. The number of unique borrowers fell from 30,033 to 7,278, and annual loans per borrower declined from 13.5 to 6.4; these annual indicators are reported in Table 2. A monthly interrupted time-series analysis of print-book loan volume (Figure 2a) used February 2020 and January 2023 as two prespecified breakpoints to examine aggregate trend changes during the pandemic and the period of GenAI diffusion, respectively; the results are reported in Table 9. Because no borrowing record was available for February 2020, that month was excluded from the regression estimation, leaving a final sample of 107 months. The model controlled for month dummy variables and used Newey–West HAC-corrected standard errors.

**Table 9:** Segmented regression results for monthly loan volume in the interrupted time-series analysis.

Parameter	Coefficient	Standard error	*t*	*p*
Baseline level (δ_0_)	36,518.078	2,630.463	13.883	< 0.001
Baseline trend/month (δ_1_)	–535.589	69.857	–7.667	< 0.001
Level change at the pandemic interruption point (δ_2_)	–9,248.807	2,813.660	–3.287	0.001
Post-pandemic slope change/month (δ_3_)	511.309	179.545	2.848	0.004
Level change at the January 2023 breakpoint (δ_4_)	1,167.392	1,748.439	0.668	0.504
Post-January 2023 slope change/month (δ_5_)	–159.558	154.033	–1.036	0.300
*R*^2^/number of sample months	0.805	*n* = 107	–	–

**Figure 2:**
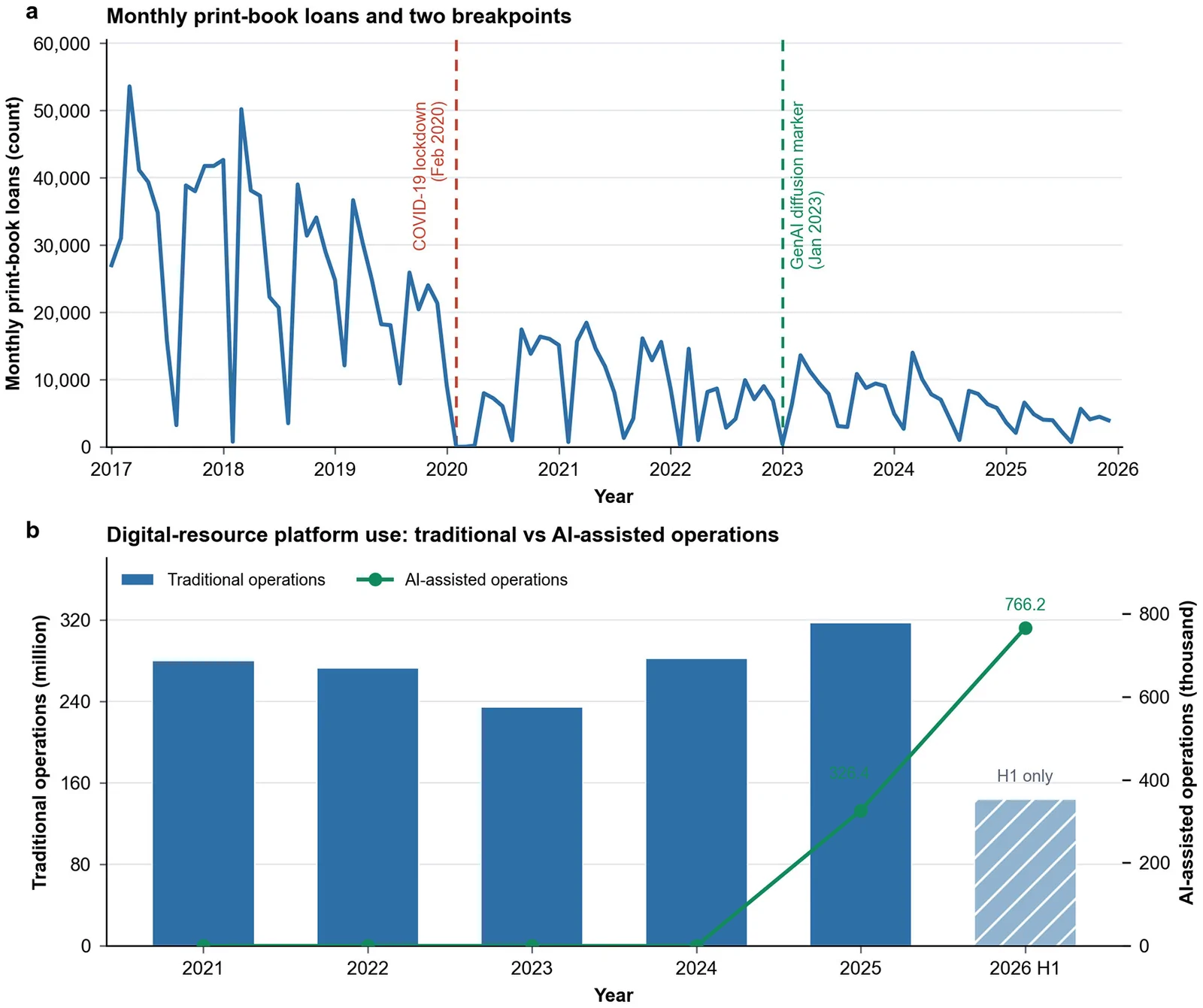
Dual-interruption trends in monthly print-book loan volume **(a)** and trends in traditional and AI-feature operations on the digital resource platform **(b)**.

Loan volume showed a long-term declining trend. Beginning in 2017, the baseline monthly trend in loan volume decreased by 535.6 books per month (*p* < 0.001). This trend preceded the emergence of GenAI.

At the pandemic interruption point, a significant level shift in loan volume was observed: the estimated change was −9,248.8 books, *p* = 0.001. The post-pandemic slope change was an increase of 511.3 books per month, *p* = 0.004, indicating that, relative to the pre-pandemic period, the decline in loan volume slowed significantly. However, after accounting for the baseline trend, the net post-pandemic trend still showed a decrease of about 24.3 books per month. During and after the pandemic, print-loan volume was significantly lower than the pre-pandemic level, consistent with service restrictions and declines in the use of physical resources reported by academic libraries worldwide during the pandemic (Mehta and Wang, 2020; Rafiq et al., 2021; Ayeni et al., 2021).

With January 2023 specified as the breakpoint, the estimated level change was 1,167.4 books (*p* = 0.504), and the slope change was −159.6 books per month (*p* = 0.300); neither was statistically significant. Sensitivity analyses replacing the GenAI interruption with March 2023, September 2023, or January 2025 (the start of the first year in which local platform statistics recorded AI-feature operations) produced the same pattern: none of the alternative level or slope changes was statistically significant (Supplementary Table S2). Across the 9 years of aggregate behavioral data, when January 2023 was specified as the breakpoint, print-loan volume showed neither a significant level change nor a significant slope change, and the pre-existing declining trend did not significantly accelerate.

#### 4.3.2 Digital resources: annual platform changes

The usage records of the digital resource platform did not show a substitution trend in which GenAI displaced the use of traditional digital resources; the relevant trend is shown in Figure 2b. Total traditional operations increased from 280 million in 2021 to 317 million in 2025, showing an overall upward trend from 2023 to 2025.

Meanwhile, the use of platform-embedded AI features grew rapidly from 2025 onward. It totaled 326,000 operations in 2025, accounting for 0.10% of total operations; in the first half of 2026 alone, it reached 766,000 operations, accounting for 0.53%. This indicates that AI features, as a new functional layer within digital resource platforms, are being progressively adopted by users rather than clearly diverting use away from traditional digital resources. The increase in AI-feature operations was not accompanied by a marked decline in traditional digital-resource operations. At the platform level, this pattern is more consistent with AI features becoming embedded in existing digital-resource services alongside traditional operations than with a clear displacement of traditional resource use. At the same time, the absolute share of AI-feature operations remains small (0.10% in 2025 and 0.53% in the first half of 2026), so these data indicate early-stage adoption of platform-embedded AI features rather than an established reconfiguration of digital-resource use.

### 4.4 Triangulation of perceptual and behavioral evidence

Integrating the questionnaire study and behavioral-data analysis, four core propositions were triangulated using two independent sources of evidence; the results are shown in Table 10.

**Table 10:** Triangulation of two independent sources of evidence.

Proposition	Perceptual evidence: questionnaire, *n* = 368	Behavioral evidence: logs and statistics	Integrated assessment
AI substitutes for digital resources	H5 was not supported, β = −0.030	Traditional digital-resource operations showed an overall upward trend	Not supported by either form of evidence
AI substitutes for print resources	H6 was supported, β = −0.100, *f*^2^ = 0.104	No significant acceleration of decline after 2023	Weakly present at the perceptual level, not yet observable at the behavioral level
Digital–AI channel integration	H2 was supported, β = 0.993	AI features are embedded in the platform, and their use continues to grow	The survey showed a positive association, but DRE and AAU lacked discriminant validity; platform data indicate only early-stage use of embedded AI features
Logic of persistence for print resources	H1 was supported, β = 0.908	A core user base remains, with 7,278 borrowers and 6.4 annual loans per borrower	The survey showed a positive association between deep-reading demand and print-resource continuance intention; borrowing records showed that print resources retained a user base

Regarding the proposition that AI substitutes for print resources, the questionnaire results show a weak negative association between substitutive GenAI use and print-resource continuance intention, whereas no corresponding change has yet been observed in aggregate-level behavioral data. This indicates that the survey-level substitution association has not yet been reflected in aggregate borrowing behavior.

## 5 Discussion

### 5.1 Main findings and theoretical dialogue

This study combines perceptual survey data and large-scale behavioral data to examine the use of academic information resources at different levels.

First, the popular narrative that “AI replaces libraries” is not supported by the data in this study. Academic information use shows a pattern of dual-channel coexistence. In the survey data, deep-reading demand was significantly and positively associated with print-resource continuance intention, with a standardized path coefficient of 0.908; the corresponding *f*^2^ met the prespecified criterion for a large effect. Auxiliary GenAI use was not significantly associated with print-resource continuance intention, whereas substitutive GenAI use showed a statistically significant but weak negative association with print-resource continuance intention. Perceived convenience of digital resources was positively associated with auxiliary GenAI use; auxiliary GenAI use was also positively associated with continuance intention toward digital resources, with a standardized path coefficient of 0.844. These findings offer empirical clues for interpreting the relationship between digital resources and auxiliary GenAI use from a complementarity perspective. These findings suggest that print and digital resources retain different bases for use, providing empirical evidence to inform channel-specific resource allocation in academic libraries. Auxiliary GenAI use can support the retrieval, screening, and comprehension of academic materials through standalone AI tools or platform-embedded functions; its positive association with continuance intention toward digital resources can be understood from a cognitive-offloading perspective as external-tool support for information processing. Substitutive GenAI use involves content production: when AI-generated text replaces students' own reading and synthesis of sources, the weak negative association between substitutive use and continuance intention toward print resources can be interpreted through the lens of deep-reading demand, whereas the association between substitutive use and continuance intention toward digital resources was not statistically significant. From a task perspective, literature retrieval and comprehension provide typical contexts for auxiliary use, whereas directly generating content for papers or reports reflects the task orientation of substitutive use. This difference in task orientation helps explain the distinct associations of the two forms of use with resource continuance intentions, although the moderating effect of task–channel fit was not statistically significant. The differential associations of these two forms of use with resource continuance intentions can be understood in terms of resource selection within self-regulated learning.

Second, digital resources and AI tools show early signs of integration. In the questionnaire data, the DRE → AAU path coefficient was 0.993, and the HTMT value for the two constructs was 0.968, indicating a close association between the two measures but insufficient discriminant validity. Platform statistics show that AI features have been embedded in digital-resource platforms and that their use has increased since 2025. The survey and platform statistics respectively capture a close self-reported association and the incorporation of AI functions into digital-resource services, suggesting early signs of integration between digital resources and AI tools (Baidoo-Anu et al., 2024; Shahzad et al., 2025), although the behavioral data show that this integration is still at an early stage: AI-feature operations accounted for only 0.10% of all platform operations in 2025 and 0.53% in the first half of 2026. We therefore interpret digital–AI integration as an emerging trend rather than an accomplished reconfiguration of academic information use. This finding also suggests that future survey instruments should more clearly distinguish perceived digital-resource convenience from auxiliary GenAI use and examine the association between them.

Third, in this model, substitutive GenAI use was negatively associated only with print-resource continuance intention, while the survey-level substitution association has not yet been reflected in aggregate borrowing behavior. In this study, substitutive GenAI use is operationalized as directly generating complete passages for papers, reports, or assignments with GenAI. Its association with print-resource continuance intention was statistically significant but weak, with a standardized path coefficient of −0.100, whereas its association with continuance intention toward digital resources was not statistically significant. Correspondingly, after 2023, print-loan volume showed neither a significant level change nor a significant slope change, and the pre-existing declining trend did not significantly accelerate. Two explanations may account for this inconsistency between the survey-level substitution association and the aggregate behavioral trend. One is social desirability bias and an attitude–behavior gap: students may overestimate the extent to which AI has changed their actual behavior. The other is a time lag: attitudinal change may precede behavioral change, and micro-level substitution may not yet have accumulated sufficiently to be detected in the institution's aggregate borrowing data. Regardless of which explanation holds, the continuing decline in print borrowing remains the more pressing issue. Print borrowing had already been declining from 2017, before the diffusion of ChatGPT, and should therefore not be attributed solely to GenAI. Students' technology-related behaviors may also be embedded within broader educational contexts, in which interpersonal and self-related factors contribute to academic engagement and achievement (Tang et al., 2026); such factors were outside the scope of the present study and were not incorporated into the proposed model. Finally, both data sources are observational. The questionnaire is cross-sectional, so the estimated paths are associations rather than causal effects; the aggregate loan series cannot identify individual-level causal effects of GenAI adoption; and the interrupted time-series design tests only whether the post-2023 period coincides with a change in the aggregate trend. Statements about “effects” throughout the manuscript should be read with these qualifications.

### 5.2 Theoretical contributions

First, this study proposes and tests an auxiliary-substitutive dual-path SEM of the associations between GenAI use and continuance intentions toward print and digital resources. It brings the two opposing positions in the literature, “AI empowers libraries” and “AI replaces libraries,” into the same analytical framework. The survey results show that auxiliary GenAI use is positively associated with continuance intention toward digital resources, whereas substitutive GenAI use shows a weak negative association with print-resource continuance intention. By distinguishing GenAI's roles in information retrieval, comprehension, and generation of complete passages for papers, reports, or assignments and interpreting the distinct associations of auxiliary and substitutive use with print-resource continuance intention and continuance intention toward digital resources through the lens of resource selection in self-regulated learning, this framework offers an educational-psychology interpretation of the divergent conclusions in prior studies.

Second, the study triangulates questionnaire evidence with 3.2 million behavioral log records and platform usage statistics, thereby providing behavioral evidence to supplement research that primarily relies on cross-sectional self-reported data. The survey-level substitution association has not yet been reflected in aggregate borrowing behavior. This discrepancy was identified by comparing perceptual and behavioral evidence.

Third, the study operationalizes the use of academic information resources in the GenAI era as a set of testable empirical propositions concerning resource-channel continuance, substitutive use, and early signs of digital–AI integration, thereby providing an analytical framework for future research on academic information services.

### 5.3 Practical implications

This study offers three implications for collection development and service redesign in academic libraries. First, H1 showed a positive association between deep-reading demand and print-resource continuance intention; print collections and space planning may therefore prioritize settings for sustained reading and systematic study. Second, H3 showed a positive association between auxiliary GenAI use and continuance intention toward digital resources; platform statistics showed that growth in AI-feature use was not accompanied by a marked decline in traditional digital-resource operations. Given that AI-feature operations still account for a small share of total platform use, libraries may consider AI search and AI-assisted reading functions in database evaluations and explore related AI-literacy education. Third, H6 showed a small negative association between substitutive GenAI use and print-resource continuance intention. Regarding the use of GenAI to generate complete passages for papers, reports, or assignments, libraries and teaching departments may provide more targeted guidance to help students distinguish auxiliary use from content substitution. This study is based on a student sample from a single research-intensive university in China and its institutional resource environment. These recommendations may serve as a reference for universities with similar resource conditions and student populations; their applicability to other universities and academic libraries requires assessment in context, and the parameter estimates should not be treated as universal benchmarks.

## 6 Limitations

Several limitations qualify the findings. First, the survey is cross-sectional and conducted at a single institution. The structural model includes use frequency as a control variable. Student status and discipline were collected but used only to describe the sample and were not included as covariates in the model; omitted-variable bias therefore cannot be ruled out. Future research could incorporate academic workload, access to resources, and digital and AI literacy to examine these associations across student groups. Second, four constructs were measured with single items, which limits the assessment of their reliability and validity. Third, DRE and AAU did not meet the discriminant-validity criteria, and common method bias cannot be ruled out; therefore, the digital-channel paths are interpreted as within-channel associations rather than causal effects. Fourth, the behavioral data are aggregate institutional records that cannot be linked to individual respondents; they provide aggregate contextual evidence for comparison with the survey findings but cannot validate the individual-level structural model. Fifth, the interrupted time-series analysis spans a period that includes the COVID-19 pandemic and its aftermath. However, the model includes an explicit COVID-19 interruption and detects no statistically significant level or slope changes under the alternative breakpoint specifications; other concurrent institutional and policy changes cannot be fully excluded. Future research should develop and validate multi-item scales for substitutive GenAI use, task–channel fit, print-resource continuance intention, and digital-resource continuance intention. It should also adopt multi-institutional, longitudinal designs with individually linked data and include direct measures of self-regulated learning, cognitive offloading, reading engagement, and learning outcomes to test further the educational-psychology interpretation advanced in this study.

## Data Availability

The datasets presented in this article are not readily available because the underlying print-circulation logs, original platform exports, and individual questionnaire responses are not deposited in a public repository because of institutional data-governance requirements, third-party platform restrictions, and participant privacy. De-identified aggregate analytical tables and analysis scripts may be available from the corresponding author upon reasonable request, subject to institutional approval, third-party permissions, and applicable privacy requirements. No public data repository is claimed. Requests to access the datasets should be directed to Jiayu Zhuang, zhuangjiayu@caas.cn.
