# Supplementary material for: Generative artificial intelligence reshapes university students' sustainable use of academic information resources: mixed evidence from behavioral logs and perceptual survey data
Source: Front Psychol. 2026 Oct 26;17:2002741. doi: 10.3389/fpsyg.2026.2002741 (PMC13745651; doi:10.3389/fpsyg.2026.2002741)
Supplement: Supplementary file 1 [file Data_Sheet_1.pdf]

Supplementary Material

1 SUPPLEMENTARY TABLES

Table S1: Questionnaire items and response coding.

Item	Analytical unit or characteristic variable	Item wording or response options
B1	Student status	Undergraduate; master's student; doctoral student; other.
B2	Disciplinary category	Humanities and social sciences; science and engineering; agriculture and medicine; economics, management, and law; arts and sports; other.
B3	Frequency of library-resource use	Almost daily; 3–5 times per week; 1–2 times per week; 2–3 times per month; 1 time per month or less.
Q1	Print reading	When I need to read deeply or systematically for study or research, I use print books.
Q2	Print reading	Even when digital resources are readily available, I still tend to use print books when sustained reading is required.
Q3	Digital access	When I need to find or browse literature quickly, I tend to use digital resource platforms.
Q4	Digital access	When I cannot visit the library or have limited time, digital resource platforms such as China National Knowledge Infrastructure (CNKI) help me search, download, or read literature online.
Q5	Task-based choice	Depending on the task, I choose among print books, digital resources, and AI tools.
Q6	AI-assisted information work	I use AI tools or AI-assisted functions in digital platforms to help me search for, screen, or locate relevant learning or research materials.
Q7	AI-assisted information work	AI tools support my reading of the literature, for example by summarizing content, explaining concepts, identifying key arguments, or organizing the structure of a text.
Q8	AI-assisted information work	I use AI tools to identify potentially relevant literature, data resources, or other research materials.
Q9	AI-assisted information work	I use AI tools to compare viewpoints, organize information, or form an initial judgment.
Q10	Generative content production	I directly use AI tools to generate complete passages or other content for papers, reports, or assignments.
Q11	Intention to continue using print resources	Even when AI tools are available, I will continue to use the library's print books.
Q12	Intention to continue using digital resources	Even when AI tools are available, I will continue to use the library's digital resource platforms.

Note: Q1–Q12 used five-point Likert-type response options, originally coded from 1 (strongly agree) to 5 (strongly disagree). The analysis reverses the coding by subtracting the original code from 6; Q5, Q10, Q11, and Q12 are analyzed as single items. AI denotes artificial intelligence.

Table S2: Sensitivity of the interrupted time-series results to alternative GenAI interruption points.

**GenAI interruption point**	**Level change (books)**	**Slope change (books/month)**	p **(level)**	p **(slope)**
January 2023 (consistent re-estimation)	1167.392	−159.558	0.504	0.300
March 2023	844.393	−173.047	0.655	0.213
September 2023	−586.541	−189.076	0.769	0.079
January 2025 (start of the first year with recorded platform AI-feature operations)	−530.358	−340.625	0.646	0.161

Note: Each row reports post-interruption level and slope changes from the interrupted time-series model of monthly print-book loan volume, re-estimated with the same specification as the main model (COVID-19 interruption in February 2020, month-of-year dummies, Newey–West HAC standard errors with a lag of 12; $n = 107$ months, February 2020 excluded). The post-interruption trend term is 0 in the interruption month and increases by 1 in each following month. The January 2023 level and slope changes match Table 9 of the main text. Under all alternative breakpoints, neither the level change nor the slope change is statistically significant at the 0.05 level.
